# Linkage between genes involved in azole resistance and ergosterol biosynthesis

**DOI:** 10.1371/journal.ppat.1008819

**Published:** 2020-09-03

**Authors:** W. Scott Moye-Rowley

**Affiliations:** Department of Molecular Physiology and Biophysics, Carver College of Medicine University of Iowa, Iowa City, Iowa, United States of America; McGill University, CANADA

Azole drugs act by blocking the biosynthesis of ergosterol, the sterol component of the fungal plasma membrane. Resistance to azole drugs appears frequently in fungi and can be typically acquired by changes in the coding sequence of the gene producing lanosterol 14α-demethylase, the azole target enzyme, or changes in the expression of this gene that is designated *ERG11* [1]. A second route of resistance involves increased expression of loci encoding ATP-binding cassette (ABC) transporters, which are thought to efflux drugs and prevent toxic intracellular concentrations from being achieved [2]. These 2 resistance mechanisms have generally been considered to represent independent, unlinked modes of lowering azole susceptibility. Recent work in several different fungi support a different viewpoint: that these resistance modalities actually illuminate a physiological tie between the biosynthesis of ergosterol and expression of ABC transporter proteins.

## Regulation of ergosterol biosynthesis and ABC transporter-encoding genes first found in *Aspergillus fumigatus*

**Fig 1:**
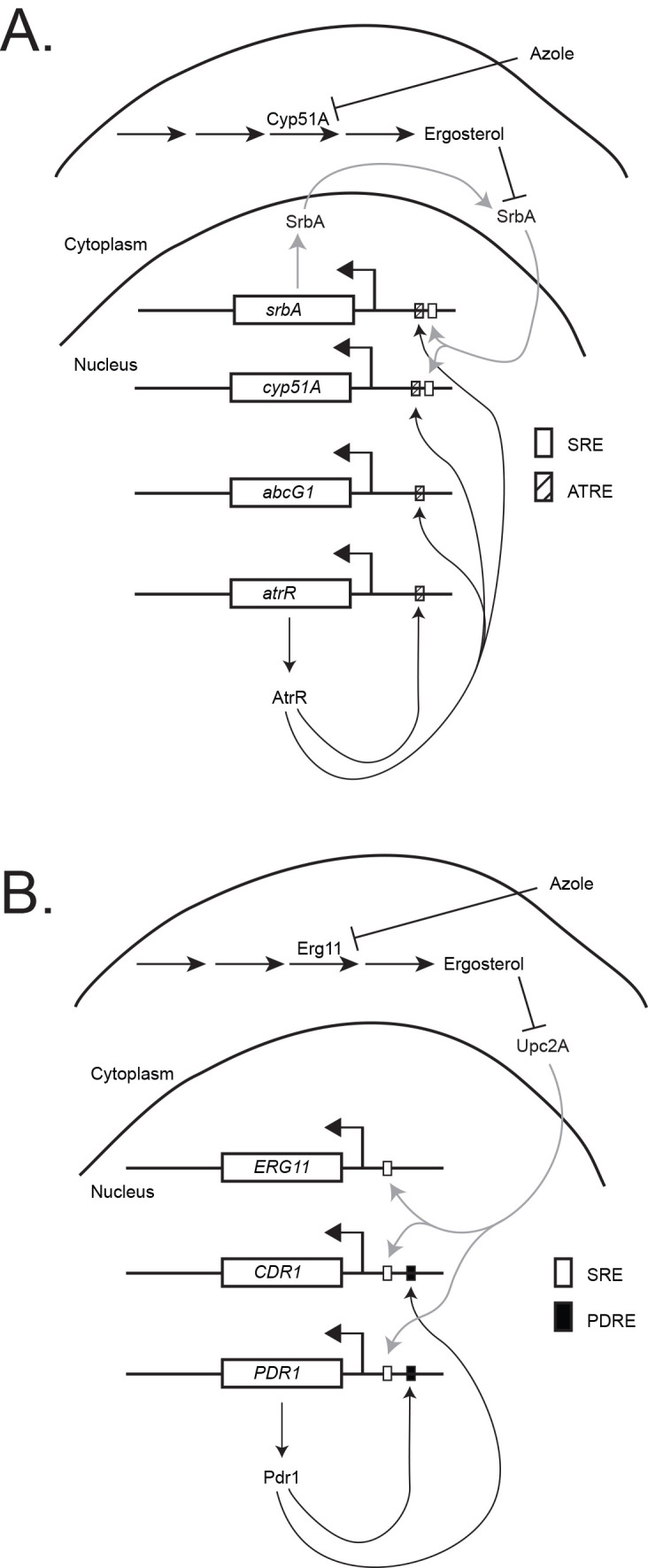
Linkage between ergosterol biosynthesis and ABC transporter expression. (A) *Aspergillus fumigatus* pathway. Ergosterol levels are depleted by azole inhibition of the Cyp51A target enzyme. This in turn leads to proteolytic activation of the SrbA transcription factor, which can then move to its target sites in the nucleus (SRE), leading to the induction of the *cyp51A* gene. We currently do not know if the AtrR transcription factor is regulated by ergosterol but this factor has been shown to induce transcription of *cyp51A*, the ABC transporter-encoding gene *abcG1*, the *srbA* gene and *atrR* itself. (B) *Candida glabrata* pathway. Depletion of ergosterol leads to an accumulation in nuclear-localized Upc2A, which, in turn, binds to the promoters of the *ERG11*, *CDR1*, and *PDR1* genes. Increased *PDR1* transcription leads to a rise in Pdr1 transcription factor levels. This triggers a further autoregulatory induction of *PDR1* expression as well as enhanced transcription of *CDR1* expression. ABC, ATP-binding cassette; SRE, sterol response elements.

The first molecular evidence linking ergosterol biosynthesis with expression of ABC transporter proteins came from studies in the filamentous fungal pathogen *Aspergillus fumigatus* [3]. It was discovered that the AtrR (ABC-transporter regulating transcription factor) transcription factor binds to the promoter of and induces the expression of *cyp51A* (the *A*. *fumigatus* ortholog of *ERG11*) as well as *abcG1*, which encodes an ABC transporter protein (Fig 1A). Work from 2 different labs had shown that *abcG1* (aka *cdr1B*) is an important determinant of azole resistance in *A*. *fumigatus* [4,5]. AtrR stimulates transcription of both of these genes, providing for coordinate transcriptional regulation. Further work determined that AtrR controls the expression of many genes in the ergosterol biosynthesis pathway and that AtrR target genes share extensive overlap with the target genes of the sterol-responsive transcription factor SrbA, including *srbA* itself [6]. Together, these data provided the first illustration of the circuitry tying expression of ergosterol biosynthetic enzymes to ABC transporters known to act on azole drugs.

Experiments in *Saccharomyces cerevisiae* had supported a model in which azole drugs can act directly as activators of transcription factors that are known to induce expression of ABC transporter-encoding genes [7]. Evidence was provided that the *S*. *cerevisiae* transcription factor Pdr1 (ScPdr1) can directly bind to ketoconazole and that this binding causes ScPdr1 to increase transcription of *PDR5*, which encodes an ABC transporter, thereby elevating drug resistance. A similar model was proposed for the azole-induced transcription in the yeast pathogen *Candida glabrata*, which contains a Pdr1 protein with high sequence conservation to ScPdr1 [8,9]. This *C*. *glabrata* protein will be referred to as CgPdr1.

These yeast data suggested that azole drugs interact with 2 intracellular proteins. They bind to the Erg11 enzyme and inhibit ergosterol synthesis, while also binding to the Pdr1 transcription factor that induces ABC transporter gene expression and stimulates azole efflux. The simplest interpretation of these data is that transcription of the ergosterol biosynthetic pathway and ABC transporters are regulated by different, independent mechanisms.

## Recent work indicates *ERG* and *PDR* genes are coregulated in *Candida glabrata*

More recent work has reevaluated this model. Two different means of genetically depleting Erg11 from *C*. *glabrata* cells were developed [10]. The *ERG11* promoter was replaced with either the methionine-repressible *MET3* promoter or a camphor-sensitive promoter that could be repressed with the addition of this terpene to the media. Inhibition of *ERG11* expression by either means led to a rapid induction of expression of Cg*PDR1*, which led to the increased expression of *CDR1*, which encodes an ABC transporter. Coupled with extensive previous data demonstrating that pharmacological inhibition of Erg11 by azole drugs led to induction of the CgPdr1/*CDR1* pathway, these genetic experiments provided evidence that the reduction of Erg11 activity was the common feature triggering activation of downstream genes, including those encoding drug efflux pumps. Although it was previously shown that azole drugs can directly bind to CgPdr1 [7], these genetic repression experiments show that direct binding is not required for activation of this transcription factor and that it can be activated by ergosterol depletion.

As was the case in *A*. *fumigatus*, further studies identified direct links between transcription factors involved in ABC transporter regulation and sterol biosynthesis in *C*. *glabrata*. The major regulator of genes encoding ergosterol biosynthetic enzymes is the transcription factor Upc2A [11]. Irrespective of whether Erg11 activity is lowered by azole drugs or genetically, induction of CgPdr1/*CDR1* requires the presence of *UPC2A* (Fig 1B) [12]. Regulation of the Upc2A homologue in *S*. *cerevisiae* (ScUpc2) has been analyzed in some detail [13]. Nuclear levels of this factor rise when ergosterol levels are depleted. Chromatin immunoprecipitation experiments suggest that this same type of regulation may occur in *C*. *glabrata* as Upc2A levels associated with Cg*PDR1* and *CDR1* rise after fluconazole treatment or genetic depletion of *ERG11* [10]. Analysis of binding to the *CDR1* promoter indicates that, even when ergosterol levels are relatively high, some pool of Upc2A can be found in the nucleus. A detailed analysis of Upc2A regulation is critical to permit understanding its role in control of target gene expression.

## Common core of fungal genes subject to co-regulation

**Fig 2:**
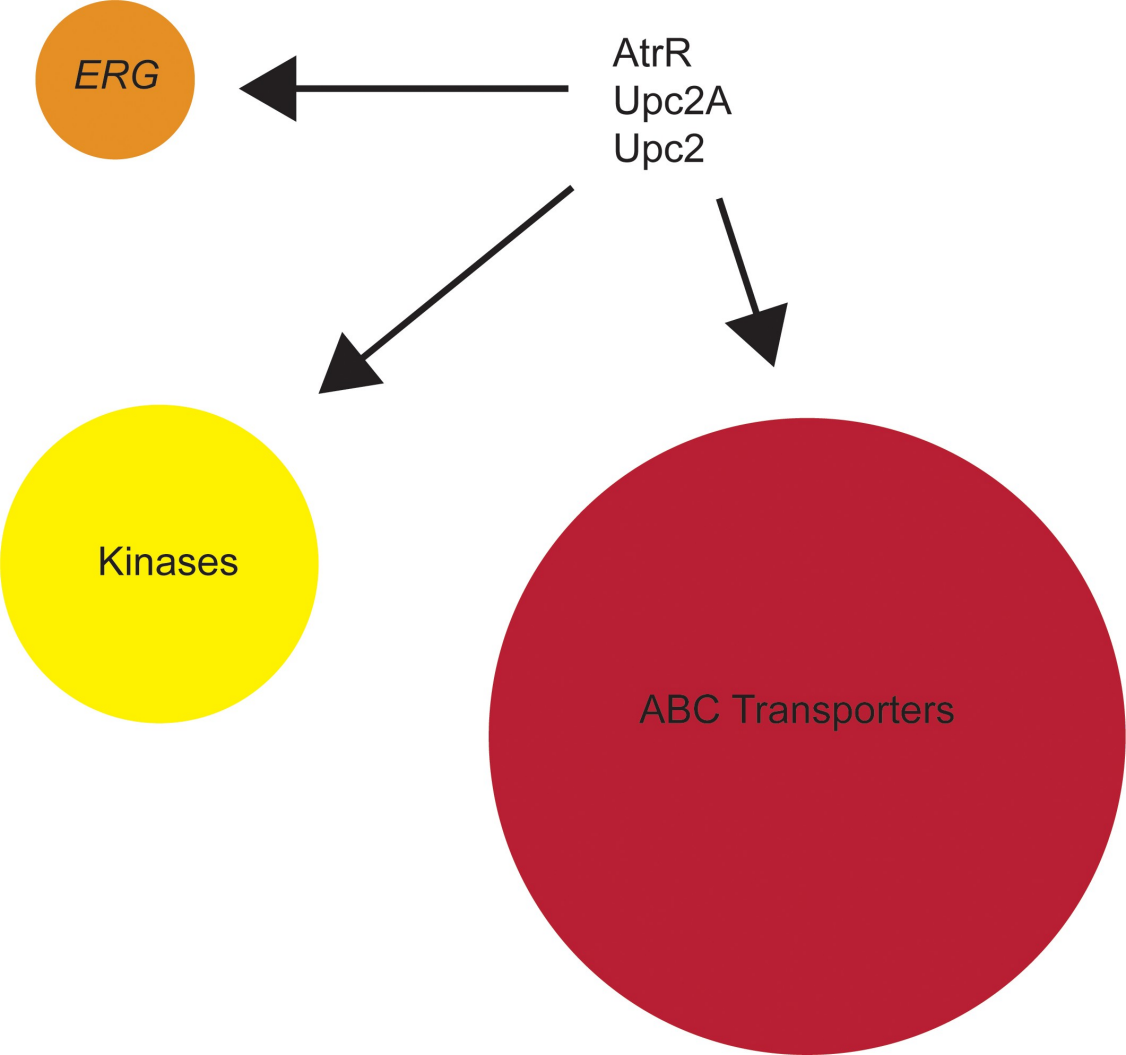
Conserved target genes across 3 fungi. Genomic binding data from *A*. *fumigatus* AtrR, *C*. *albicans* Upc2, and *C*. *glabrata* Upc2A was converted to nonsyntenic homologues from *S*. *cerevisiae*. Homologues that were identified in all 3 fungi were grouped by protein classes shown. The circles are scaled proportionately to the number of homologous genes identified.

While the details underlying organism-specific linkage of expression of ABC transporter proteins and ergosterol biosynthesis are different, an overarching question is why does this linkage exist? Using available ChIP-seq data from *A*. *fumigatus* AtrR [6], *C*. *albicans* Upc2 [14] and ChIP-seq data from *C*. *glabrata* Upc2A (B. Vu and M. Stamnes, Personal Communication), I converted the genes from each fungus into their nonsyntenic *S*. *cerevisiae* homologues. This allows comparison of the different suites of targets genes from each organism. This comparison is shown in Fig 2.

There are 3 main types of genes that are predicted to be coregulated by all 3 fungal transcription factors. These common target genes encode multiple ABC transporters, 3 enzymes in the ergosterol biosynthetic pathway (*ERG1*, *ERG11*, and *ERG25*) as well as several proteins with kinase activity. The ABC transporters include 7 involved in drug resistance and found at the plasma membrane (Pdr5, Pdr11, Pdr12, Pdr15, Pdr18, Snq2, and Yor1), 2 involved in sterol import (Pdr11 and Aus1) and 5 localized to the vacuole (Bpt1, Nft1, Ybt1, Ycf1, and Vmr1). The kinase enzymes consist of 4 sugar kinases (Glk1, Emi2, Hxk1, and Hxk2) and 2 protein kinases (Sks1 and Vhs1). While the nonsyntenic nature of their conservation identifies a wide range of homologues, these conserved genes suggest links between membrane transport events, metabolism, and protein phosphorylation. Even across these very different fungi, this common core of genes is transcriptionally tied together via common regulatory factors. This emphasizes the likely biological importance of coordinating the expression of these genes.

## Proper plasma membrane function may underlie coregulatory network

A potential explanation for this coordinate control of gene expression is that it represents a common response to plasma membrane stress events. It is well-established that ABC transporters control the distribution of the lipid contents of membranes [15]. Changes in ergosterol biosynthesis would certainly impact membrane composition, and protein kinases can provide an additional regulatory input. Accumulation of ergosterol intermediates could also trigger activation of ABC transporter-encoding genes to prevent any toxic effects from buildup of ergosterol biosynthetic intermediates. It is also striking that the 3 most commonly regulated *ERG* pathway genes are all involved in oxygen-dependent reactions that convert squalene to ergosterol [16]. Mutants lacking either Upc2 homolog in *Candida* species or *atrR* in *A*. *fumigatus* [3] have already been shown to be defective in hypoxic growth, but the linkage between ABC transporters and low oxygen levels is less clear at this time.

It is possible that coordinate transcriptional control of ergosterol biosynthesis and ABC transporter protein production is required to ensure the normal function of the plasma membrane as the primary barrier dividing the intracellular milieu from the extracellular environment. Plasma membrane function is critically determined by its lipid and protein components. Ergosterol is a key modulator of membrane fluidity [17] and changes in this property of the plasma membrane is both monitored [18] and can trigger a range of physiological responses (reviewed in [19]). It is certainly possible that the action of the plasma membrane ABC transporters (like Cdr1 in Candida species) interact with sterols and act as the direct modulators of decisions made to permit accumulation of various compounds in the cell.

Another possibility is that sterols and ABC transporters act indirectly to modulate drug resistance through their control of the lipid composition of the plasma membrane, in which a large number of other transporter proteins reside. Coupled with the implication of fungal ABC transporters as regulators of lipid content [20], another model that would fit all available data is that ergosterol levels and ABC transporters function as indirect regulators of drug efflux. The large number of membrane transporters including and beyond the ABC class might influence drug efflux with their activity being modulated by the lipid content controlled by ergosterol levels and ABC transporters.

## Future prospects

Irrespective of which explanation ultimately explains the coordinate regulation of ergosterol biosynthesis and ABC transporter expression, the study of azole resistance in fungi has uncovered a new tie between lipid production and membrane protein biosynthesis. Understanding this tie will provide new insight into both drug resistance and also membrane biology. Furthermore, since 2 of the 3 main antifungal drugs target ergosterol biosynthesis, analysis of this connection will likely shed new light on processes sensitive to chemotherapeutic interventions.
